# Iterative Tomographic Image Reconstruction Algorithm Based on Extended Power Divergence by Dynamic Parameter Tuning

**DOI:** 10.3390/jimaging10080178

**Published:** 2024-07-23

**Authors:** Ryuto Yabuki, Yusaku Yamaguchi, Omar M. Abou Al-Ola, Takeshi Kojima, Tetsuya Yoshinaga

**Affiliations:** 1 Graduate School of Health Sciences, Tokushima University, 3-18-15 Kuramoto, Tokushima 770-8509, Japan; c202322008@tokushima-u.ac.jp; 2 Shikoku Medical Center for Children and Adults, National Hospital Organization, 2-1-1 Senyu, Zentsuji 765-8507, Japan; yamaguchi.yusaku.sf@mail.hosp.go.jp; 3 Faculty of Science, Tanta University, El-Giesh St., Tanta 31527, Egypt; omr.aboualaela@science.tanta.edu.eg; 4 Institute of Biomedical Sciences, Tokushima University, 3-18-15 Kuramoto, Tokushima 770-8509, Japan; yosinaga@medsci.tokushima-u.ac.jp

**Keywords:** extended power divergence, computed tomography, iterative reconstruction, optimization, dynamic parameter tuning

## Abstract

Computed tomography (CT) imaging plays a crucial role in various medical applications, but noise in projection data can significantly degrade image quality and hinder diagnosis accuracy. Iterative algorithms for tomographic image reconstruction outperform transform methods, especially in scenarios with severe noise in projections. In this paper, we propose a method to dynamically adjust two parameters included in the iterative rules during the reconstruction process. The algorithm, named the parameter-extended expectation-maximization based on power divergence (PXEM), aims to minimize the weighted extended power divergence between the measured and forward projections at each iteration. Our numerical and physical experiments showed that PXEM surpassed conventional methods such as maximum-likelihood expectation-maximization (MLEM), particularly in noisy scenarios. PXEM combines the noise suppression capabilities of power divergence-based expectation-maximization with static parameters at every iteration and the edge preservation properties of MLEM. The experimental results demonstrated significant improvements in image quality in metrics such as the structural similarity index measure and peak signal-to-noise ratio. PXEM improves CT image reconstruction quality under high noise conditions through enhanced optimization techniques.

## 1. Introduction

Computed tomography [1,2,3,4] (CT) imaging plays a crucial role in medical diagnostics by producing the detailed tomographic images necessary for accurate diagnosis and treatment planning. The problem of image reconstruction in CT can be reduced to an optimization problem of finding pixel values that minimize the difference between measured and forward projections. Iterative methods [5,6,7,8,9] for solving optimization problems provide high-quality images for ill-posed inverse problems compared with transform methods [1,2]. Many iterative reconstruction methods based on the minimization principle are known, such as the algebraic reconstruction technique [5], maximum-likelihood expectation-maximization (MLEM) method [10], and multiplicative algebraic reconstruction technique [11,12,13]. Iterative algorithms that yield reconstructions with fewer artifacts and a higher resolution compared with traditional methods, even under conditions such as high noise levels in projections [14] or a limited number of projections [15], are actively being developed. Recent advances in CT image reconstruction, driven by machine-learning techniques [16,17], have markedly improved quality and efficiency by learning complex patterns in data. However, challenges persist in handling diverse patient data and adapting to variable scan conditions, highlighting the need for novel optimization strategies. This study introduces an iterative reconstruction algorithm with dynamic parameter tuning, aiming to enhance image fidelity while optimizing computational efficiency for clinical use.

The performance of algorithms, in terms of convergence or image quality, depends on the objective function [18,19,20,21] that decreases the differences iteratively. An iterative algorithm using the extended power divergence [22] (EPD) as an objective function has been proposed. This algorithm, referred to as the power divergence-based expectation-maximization (PDEM) method, provides higher convergence performance and better-quality reconstructions compared with the MLEM method by choosing appropriate constant values of two parameters throughout the iteration process. One way to determine parameter values that yield effective results is to use machine learning [23], but this requires pre-training on various imaging subjects and noise levels.

In this paper, as an improvement to the PDEM algorithm, we propose a method to provide different parameter values for each iteration. Conventional iterative reconstruction algorithms often include relaxation parameters such as those in the algebraic reconstruction technique (ART) or simultaneous ART, and methods have been proposed [24,25,26] to adaptively adjust the values of these relaxation parameters based on projection data and the noise level. If we regard iterative algorithms as numerical discretizations of systems of differential equations [27,28], then the relaxation parameters correspond to the step size of an additive or multiplicative discretization, and changes in step size essentially affect the computational accuracy and speed of the system. On the other hand, the two parameters of the PDEM algorithm directly affect the quality of the reconstructed images, and adjusting the parameter values appropriately for each iteration can be highly effective. The strategy to determine the parameter values of the PDEM method utilizes the optimization principle of the evaluation function. However, performing extensive optimization operations to adjust parameters at each iteration is impractical. Therefore, we replace the target system with a reduced-size one to calculate parameter sequences for each iteration in advance and then apply the PDEM algorithm with the re-adjusted parameters for each iteration to the original target system to achieve the same effect as an iterative adjustment. Here, we conducted numerical experiments comparing the reconstructed images with the original images under the assumption of projection noise by using not only an evaluation function of the measured and forward projections but also the multi-scale structural similarity index measure [29] (MS-SSIM) and peak signal-to-noise ratio (PSNR). The following results were obtained: The parameter sequences of the original and downsized systems were nearly identical, and it was found that the images reconstructed by the proposed algorithm exhibited higher quality in the low-frequency and high-frequency image regions compared with those reconstructed using MLEM and PDEM. Moreover, since the proposed algorithm inherently incorporates the principle of optimizing the evaluation function value at each iteration, it outperforms other methods under the same evaluation function, even at any iteration number.

By constructing an iterative image reconstruction method using dynamic parameter tuning based on optimization principles, it is possible to achieve both the suppression of measurement noise and preservation of image edges. The proposed method is expected to contribute to the principles of CT scanner reconstruction by producing high-quality images even under conditions with relatively high projection noise.

The remainder of this paper is organized as follows: Section 2 describes the proposed method. Specifically, Section 2.1 defines EPD and theoretically examines its properties, while Section 2.2 explains the iterative algorithm with dynamic parameter tuning that we propose. Section 2.3 introduces ideas to shorten the computation time. Section 3 validates the proposed method, detailing the experimental methods in Section 3.1 and discussing the experimental results in Section 3.2. Finally, Section 4 presents the conclusions of this paper.

## 2. Proposed Method

In this section, we provide the definition of EPD, which plays an important role in both the reconstruction principle and parameter optimization of the proposed algorithm. Then, after briefly illustrating its properties, we present an algorithm for the proposed method and a practical strategy for its implementation.

### 2.1. Definition and Properties of EPD

For given parameters γ>0 and α≥0, the EPD between positive values *p* and *q* is defined as   

(1) $$\phi_{\gamma ,\alpha }\left(p,q\right)=\int_{p}^{q}\frac{s^{\gamma }-p^{\gamma }}{s^{\gamma \alpha }}ds$$

which can be expressed as

$$\begin{array}{ccc} \phi_{\gamma ,\alpha }\left(p,q\right) & = & \int_{p}^{q}\frac{s^{\gamma }-p^{\gamma }}{s}ds\\ & = & \frac{1}{\gamma }p^{\gamma }\left(\log{\left(\frac{p}{q}\right)}^{\gamma }+{\left(\frac{q}{p}\right)}^{\gamma }-1\right) \end{array}$$

if 1−γα=0,

$$\begin{array}{ccc} \phi_{\gamma ,\alpha }\left(p,q\right) & = & \int_{p}^{q}\frac{s^{\gamma }-p^{\gamma }}{s^{1+\gamma }}ds\\ & = & \frac{1}{\gamma }\left(\log{\left(\frac{q}{p}\right)}^{\gamma }+{\left(\frac{p}{q}\right)}^{\gamma }-1\right) \end{array}$$

if 1+γ(1−α)=0, and

$$\begin{array}{ccc} \phi_{\gamma ,\alpha }\left(p,q\right) & = & \int_{p}^{q}\frac{s^{\gamma }-p^{\gamma }}{s^{\gamma \alpha }}ds\\ & = & \frac{1}{1-\gamma \alpha }p^{1+\gamma (1-\alpha )}\left(1-{\left(\frac{q}{p}\right)}^{1-\gamma \alpha }\right)\\ & & \;+\frac{1}{1+\gamma (1-\alpha )}p^{1+\gamma (1-\alpha )}\left({\left(\frac{q}{p}\right)}^{1+\gamma (1-\alpha )}-1\right) \end{array}$$

otherwise. In the special case where either *p* or *q* equals 0, we define as follows: when p=0,

(2) $$\phi_{\gamma ,\alpha }\left(0,q\right)=\frac{1}{1+\gamma (1-\alpha )}q^{1+\gamma (1-\alpha )}$$

if 1+γ(1−α)>0 and

(3) $$\phi_{\gamma ,\alpha }\left(0,q\right)=\infty$$

otherwise; whereas, when q=0,

(4) $$\phi_{\gamma ,\alpha }\left(p,0\right)=\frac{\gamma }{\left(1-\gamma \alpha )(1+\gamma (1-\alpha )\right)}p^{1+\gamma (1-\alpha )}$$

if 1−γα>0 (thus, 1+γ(1−α)>0) and

(5) $$\phi_{\gamma ,\alpha }\left(p,0\right)=\infty$$

otherwise.

The value of EPD is zero if p=q and positive if p≠q because the integrand

$$\frac{s^{\gamma }-p^{\gamma }}{s^{\gamma \alpha }}=\left(1-{\left(\frac{p}{s}\right)}^{\gamma }\right)s^{\gamma (1-\alpha )}$$

in Equation (1) for positive values of *p* and *q* over the interval [p,q] is nonnegative, owing to the fact that ${\left(p/s\right)}^{\gamma }$ is less than or equal to 1 and $s^{\gamma (1-\alpha )}$ is positive. Positivity in the case where either *p* or *q* is zero is also ensured by Equations (3) and (5).

Under certain conditions, the EPD can be shown to provide upper bounds for well-known functions.

**Proposition** **1.** *Assume that p and q are in the range [0,1]. Then, the inequality*

$$\phi_{\gamma ,\alpha }\left(p,q\right)\geq \phi_{\gamma ,1}\left(p,q\right)$$

*is satisfied for γ>0 and α≥1.*

**Proof.**  We have

$$\begin{array}{ccc} \phi_{\gamma ,\alpha }\left(p,q\right)-\phi_{\gamma ,1}\left(p,q\right) & = & \int_{p}^{q}\left(1-{\left(\frac{p}{s}\right)}^{\gamma }\right)\left(s^{\gamma (1-\alpha )}-1\right)ds\\ & \geq & 0 \end{array}$$

because $s^{\gamma (1-\alpha )}\geq 1$ holds for γ>0, α≥1, and s∈[0,1].    □

Note that $\phi_{\gamma ,1}\left(p,q\right)$ indicates the Kullback–Leibler (KL) divergence [30] and Hellinger distance when the values of γ are 1 and 0.5, respectively. Moreover, it is known that the KL-divergence upper-bounds the squared total variation. Hence, with appropriate parameters, the extended power divergence minimizes the KL-divergence, squared total variation, and Hellinger distance as well.

### 2.2. Iterative Reconstruction Algorithm by Dynamic Parameter Tuning

The relationship between the projections is $y\in R_{+}^{I}$, where $R_{+}$ represents the set of nonnegative real numbers, obtained from a CT scanner, and the reconstructed image $x\in R_{+}^{J}$ is assumed to be

(6) y=Ax+σ,

where $A\in R_{+}^{I\times J}$ is the projection operator, and $\sigma \in R_{+}^{I}$ is noise. Iterative image reconstruction involves a suitable number *N* of iterations for a predetermined algorithm, starting from an initial image $z\left(0\right)=x^{0}$. An image z(n+1) is generated at each iteration n=0,1,2,…,N−1, and the final reconstructed image *x* is z(N).

Our iterative image reconstruction algorithm is defined as follows, by using γ(n+1) and α(n+1) as parameters that change according to the number of iterations and are precomputed by the method described later:

(7) $$z_{j}\left(n+1\right)=f_{j}\left(z\left(n\right),\gamma \left(n+1\right),\alpha \left(n+1\right)\right)$$

for j=1,2,…,J and n=0,1,2,…,N−1, where

(8) $$f_{j}\left(z,\gamma ,\alpha \right):=z_{j}\frac{\sum_{i=1}^{I}a_{ij}{\left(\frac{y_{i}}{{\left(A_{i}z\right)}^{\alpha }}\right)}^{\gamma }}{\sum_{i=1}^{I}a_{ij}{\left(\frac{A_{i}z}{{\left(A_{i}z\right)}^{\alpha }}\right)}^{\gamma }}$$

with $z_{j}$, $y_{i}$, $A_{i}$ and $a_{ij}$ being the *j*th element of *z*, *i*th element of *y*, *i*th row vector of *A*, and the element at the *i*th row and *j*th column of *A*, respectively, for i=1,2,…,I and j=1,2,…,J. For a fixed set of parameters $\left(\gamma^{0},\alpha^{0}\right)$ and a given state variable z(n) at the *n*th iteration, the following optimization problem is solved for the set of parameters (γ(n+1),α(n+1)) in Equation (7) for n=0,1,2,…,N−1:

(9) $$\left(\gamma \left(n+1\right),\alpha \left(n+1\right)\right)=\underset{\gamma >0,\;\alpha \geq 0}{\mathrm{argmin}}\Phi \left(z\left(n\right),\gamma ,\alpha \right)$$

where

(10) $$\Phi \left(z\left(n\right),\gamma ,\alpha \right):=\sum_{i=1}^{I}\phi_{\gamma^{0},\alpha^{0}}\left(y_{i},A_{i}f\left(z\left(n\right),\gamma ,\alpha \right)\right)\sum_{j=1}^{J}a_{ij}$$

with *f* denoting the vector whose *j*th element is $f_{j}$ for j=1,2,…,J. Note that, when the parameters do not depend on *n* and are predetermined constants, Equation (7) is identical to the equation of the PDEM algorithm presented in Ref. [22]. Furthermore, when the parameter set (γ,α) equals (1,1), Equation (7) corresponds to MLEM. When searching for a set of parameters (γ,α) that minimize the objective function Φ, the optimization problem initialized with (γ(n),α(n)) is solved in order to find (γ(n+1),α(n+1)) for each n=0,1,2,…,N−1. (γ(0),α(0)) is given as a fixed value; for example, (1,1). Algorithm 1 lists pseudocode for the proposed method, referred to as ‘parameter-extended expectation-maximization based on power divergence’ (PXEM).
**Algorithm 1** Procedure of PXEM algorithm**Require:** $y\in R_{+}^{I}$, $A\in R_{+}^{I\times J}$, $x^{0}\in R_{+}^{J}$, N>0, $\gamma^{0}>0$, $\alpha^{0}\geq 0$   1:(γ(0),α(0))←(1,1)   2:$z\left(0\right)\leftarrow x^{0}$   3:n←0   4:**while** n<N **do**   5:    $\left(\gamma \left(n+1\right),\alpha \left(n+1\right)\right)\leftarrow \underset{\gamma >0,\;\alpha \geq 0}{\mathrm{argmin}}\sum_{i=1}^{I}\phi_{\gamma^{0},\alpha^{0}}\left(y_{i},A_{i}f\left(z\left(n\right),\gamma ,\alpha \right)\right)\sum_{j=1}^{J}a_{ij}$   6:    z(n+1)←f(z(n),γ(n+1),α(n+1))   7:    n←n+1   8:**end while****Return:** z(N)

The algorithm using PDEM aims to decrease the objective function of the image *z* iteratively,

(11) $$V\left(z\right):=\sum_{i=1}^{I}\phi_{\gamma ,\alpha }\left(y_{i},A_{i}z\right),$$

which is the element-wise sum of the EPDs between the measured and forward projections. Since *V* coincides with the generalized KL-divergence when γ=1 and α=1, PDEM becomes an iterative algorithm extended from the MLEM by two parameters. It has been experimentally demonstrated [22] that, when projections contain a significant amount of noise, PDEM yields higher-quality images with less noise compared with those reconstructed by MLEM. Since the parameters of PDEM are fixed, the procedure follows the same iterative algorithm as usual, but, for comparison with PXEM, we have outlined the process in Algorithm 2.
**Algorithm 2** Procedure of PDEM algorithm**Require:** $y\in R_{+}^{I}$, $A\in R_{+}^{I\times J}$, $x^{0}\in R_{+}^{J}$, N>0, γ>0, α≥0   1:$z\left(0\right)\leftarrow x^{0}$   2:n←0   3:**while** n<N **do**   4:    z(n+1)←f(z(n),γ,α)   5:    n←n+1   6:**end while****Return:** z(N)

### 2.3. Practical Strategy for Dynamic Parameter Tuning Using Reduced-Size System

The strategy presented below addresses the computational overhead associated with the optimization process, and we will refer to this method as ‘projected reduced-system expectation-maximization based on power divergence’ (PREM).

The image with a pixel number of *J* is assumed to be a square image with sides of $J_{s}$, and the number of projections *I* is the product of the number of bins $I_{b}$ and the number of projection directions $I_{p}$. Furthermore, assuming a common multiple *M* exists for $J_{s}$ and $I_{p}$, we define ${\bar{J}}_{s}$ and ${\bar{I}}_{p}$ such that $J_{s}={\bar{J}}_{s}\times M$ and $I_{p}={\bar{I}}_{p}\times M$. In this case, we consider the following system of reduced pixel number and projection number: $\bar{J}={\bar{J}}_{s}\times {\bar{J}}_{s}$ and $\bar{I}={\bar{I}}_{b}\times {\bar{I}}_{p}$, where ${\bar{I}}_{b}$ is a natural number determined by ${\bar{J}}_{s}$ that represents the number of bins. Here, because the scale of the data required for operations is the product of the pixel number and projection number, the reduction is carried out in order of the inverse of $M^{4}$.

The procedure of using the reduced system is as follows: First, interpolate the projections of the given image reconstruction system on the sinogram plane to obtain the reduced system’s projections. Next, determine the parameters for each iteration up to the target maximum iteration number from the obtained projections and the projection operators of the reduced system. Finally, apply the PDEM algorithm with the parameters set for each iteration using the obtained parameter sequence to the original image reconstruction system. Algorithm 3 lists the pseudocode of PREM. Here, the reconstruction dataset for the reduced system $\bar{f}$ corresponding to *f* in Equation (8) with respect to the original reconstruction dataset $\left\{y\in R_{+}^{I},A\in R_{+}^{I\times J},x^{0}\in R_{+}^{J}\right\}$ is denoted as $\left\{\bar{y}\in R_{+}^{\bar{I}},\bar{A}\in R_{+}^{\bar{I}\times \bar{J}},{\bar{x}}_{0}\in R_{+}^{\bar{J}}\right\}$.
**Algorithm 3** Procedure of PREM algorithm**Require:** $y\in R_{+}^{I}$, $A\in R_{+}^{I\times J}$, $x^{0}\in R_{+}^{J}$, $\bar{y}\in R_{+}^{\bar{I}}$, $\bar{A}\in R_{+}^{\bar{I}\times \bar{J}}$, ${\bar{x}}^{0}\in R_{+}^{\bar{J}}$, N>0, $\gamma^{0}>0$, $\alpha^{0}\geq 0$   1:(γ(0),α(0))←(1,1)   2:$\bar{z}\left(0\right)\leftarrow {\bar{x}}^{0}$   3:n←0   4:**while** n<N **do**   5:    $\left(\gamma \left(n+1\right),\alpha \left(n+1\right)\right)\leftarrow \underset{\gamma >0,\;\alpha \geq 0}{\mathrm{argmin}}\sum_{i=1}^{\bar{I}}\phi_{\gamma^{0},\alpha^{0}}\left({\bar{y}}_{i},{\bar{A}}_{i}\bar{f}\left(\bar{z}\left(n\right),\gamma ,\alpha \right)\right)\sum_{j=1}^{\bar{J}}{\bar{a}}_{ij}$   6:    $\bar{z}\left(n+1\right)\leftarrow \bar{f}\left(\bar{z}\left(n\right),\gamma \left(n+1\right),\alpha \left(n+1\right)\right)$   7:    n←n+1   8:**end while**   9:$z\left(0\right)\leftarrow x^{0}$ 10:n←0 11:**while** n<N **do** 12:    z(n+1)←f(z(n),γ(n+1),α(n+1)) 13:    n←n+1 14:**end while****Return:** z(N)

## 3. Experiment

We conducted numerical and physical experiments on the image reconstruction methods using the PXEM and PREM algorithms with the dynamic parameter tuning proposed in Section 2.2 and Section 2.3.

### 3.1. Experimental Method

**Figure 1:**
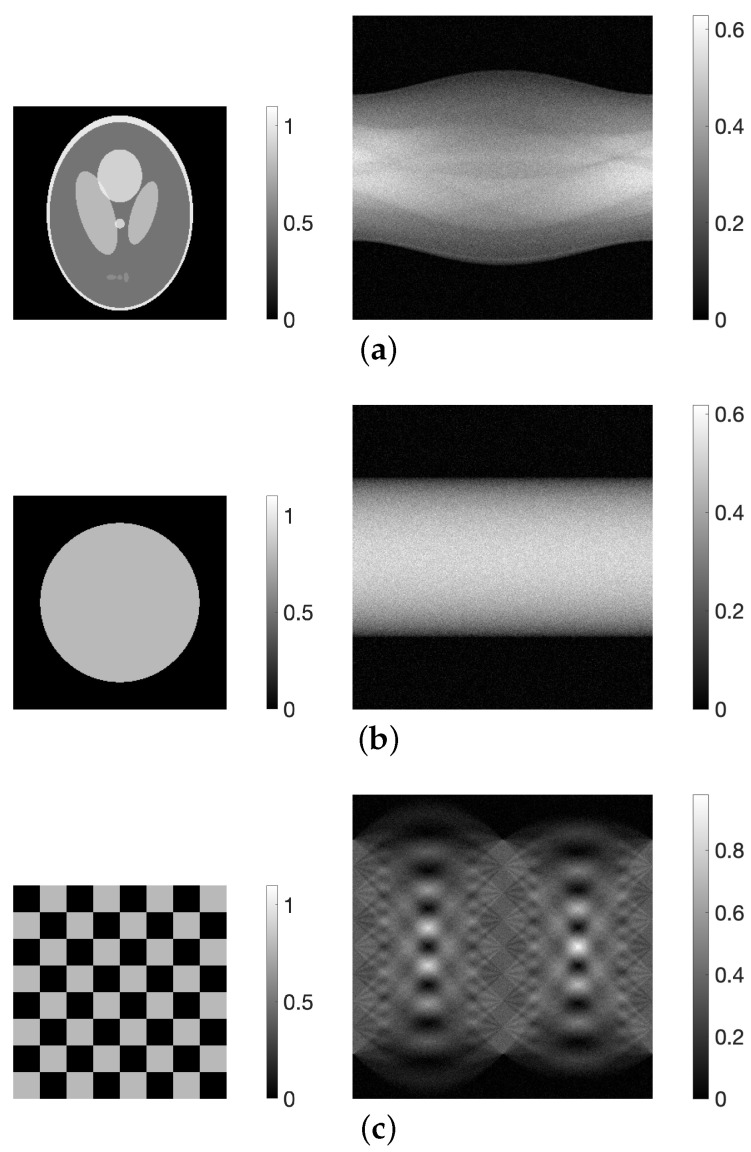
(**a**) Modified Shepp–Logan, (**b**) disc, and (**c**) chessboard digital phantoms (**left**) and their sinograms (**right**).

For the numerical experiments, we utilized three types of phantom: the modified Shepp–Logan [31], disc, and chessboard, as shown in Figure 1. Each image $e\in R_{+}^{J}$ had a size of 256×256 (J=65,536), with pixel values ranging from 0 to 1. Projections $y\in R_{+}^{I}$ were simulated with 180-degree sampling, resulting in 360 projection directions and 365 detectors (I=131,400). White noise with a signal-to-noise ratio (SNR) of 20 dB was added to the projections. This was done because previous studies [22] have shown that high-quality images with reduced noise effects compared with MLEM images can be obtained by providing appropriate parameters to the PDEM method when the noise level is relatively high.

**Figure 2:**
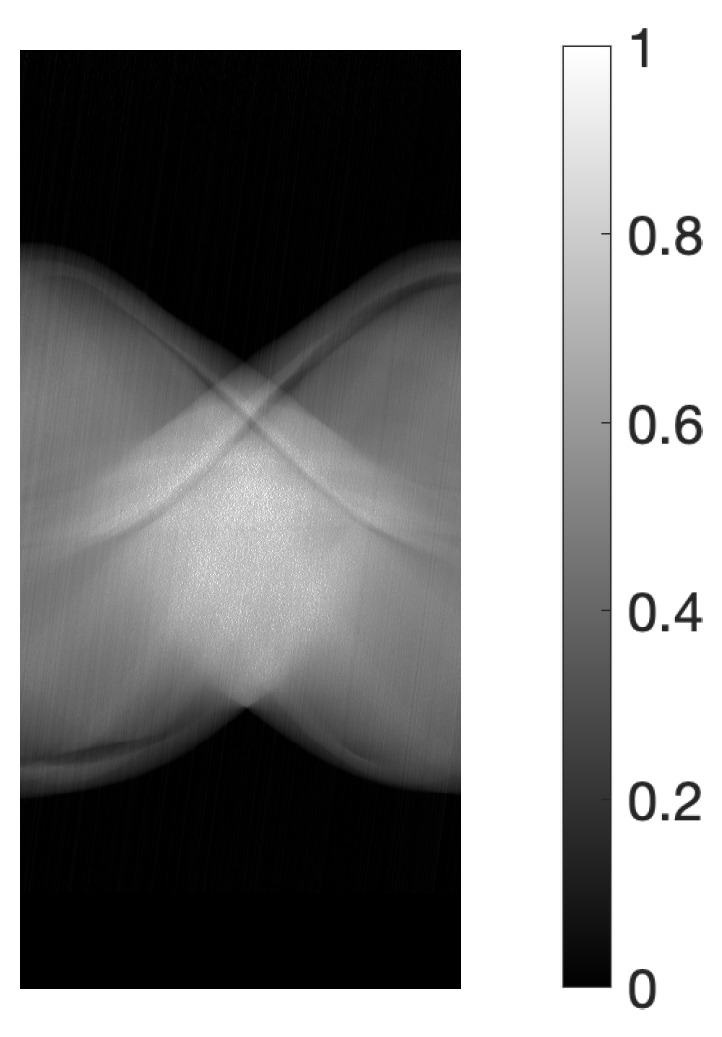
Sinogram obtained from X-ray CT scanner.

For the physical experiments, the projections were obtained using an X-ray CT scanner (Canon Medical Systems, Tochigi, Japan) and a body-simulated phantom [32] (Kyoto Kagaku, Kyoto, Japan). The scanner was operated at an 80 kVp tube voltage and 30 mA tube current, with an exposure time of 0.75 s per rotation. This setting pertains to conditions where imaging is conducted under relatively high levels of measurement noise. Figure 2 illustrates the sinogram, a two-dimensional array of data containing the projections $y\in R_{+}^{I}$, where I=430,650 (957 acquisition bins and 450 projection directions spanning 180 degrees). The size of the reconstructed image was 675×675 (J=455,625).

The initial value for the iterations was set to

(12) $$x_{j}^{0}=\sum_{i=1}^{I}y_{i}/\sum_{i=1}^{I}\sum_{k=1}^{J}a_{ik}$$

for j=1,2,…,J as in the MLEM method, and the iterations ranged up to a maximum of N=30 in all experiments. The MATLAB (MathWorks, Natick, MA, USA) function fmincon, which seeks the minimum of a constrained nonlinear multivariable function, was used to solve Equation (9) numerically. The initial values of the parameters were (γ(0),α(0))=(1,1). The lower limit of the parameter variation was set to 0, while the upper limit was set to 1.4 in the numerical experiments and 2.0 in the physical experiments. Setting an upper limit is necessary to ensure the accuracy of the numerical computations.

To satisfy the conditions of Proposition 1, in the numerical experiments, the projections were set to be within the range [0,1], and in the physical experiments, the projections were normalized so that the maximum value would be 1, as shown in Figure 1 and Figure 2. In the numerical experiments, the size of the reduced system used in PREM was set so as to ensure M=4, with ${\bar{J}}_{s}=64$ and ${\bar{I}}_{p}=90$. In the physical experiments, the size was adjusted to M=3, resulting in ${\bar{J}}_{s}=225$ and ${\bar{I}}_{p}=150$.

Moreover, in the numerical experiments, to compare the reconstructed image *x* with the true image *e*, we utilized the MS-SSIM with the same set of scale weights as in Ref. [29] and the PSNR between the true and reconstructed images. To examine the effectiveness of the parameter optimization described by Equation (9), we defined the evaluation function E(z) of the reconstructed image *z* as follows:

(13) $$E\left(z\left(n\right)\right)=\sum_{i=1}^{I}\phi_{\gamma^{0},\alpha^{0}}\left(y_{i},A_{i}z\left(n\right)\right)\sum_{j=1}^{J}a_{ij}$$

for n=0,1,2,…,N, which is a weighted extended power divergence between the measured and forward projections. We used this function in both the numerical and physical experiments. The parameters $\left(\gamma^{0},\alpha^{0}\right)$ of the PXEM and PREM methods in Equations (9), (10), and (13) were fixed at (0.5,1.2). The fixed parameters in the PDEM method were set to the same values (γ,α)=(0.5,1.2). This set of parameters is based on the numerical analysis results in Ref. [22], which proposed the PDEM algorithm. It has been confirmed to provide good performance in numerical experiments, assuming projection noise. Even in physical experiments where the noise level cannot be accurately detected, good results have been obtained using the same parameter values. However, the optimal values vary depending on the subject of the imaging and the noise level, and this issue motivated the development of a solution for parameter tuning in each iteration in this study. The same values for (γ,α) were used for $\left(\gamma^{0},\alpha^{0}\right)$ to match the evaluation function used for the results with the optimization function used to derive the reconstruction algorithm of PDEM.

For the numerical computations, we utilized a computer equipped with an eight-core Intel Xeon processor clocked at 3.5 GHz and 98 GB of memory. The computational time required for the reconstruction experiment was within a few minutes, even for large-scale physical experiments. Specific computational times are presented in the experimental results.

### 3.2. Experimental Results and Discussion

**Figure 3:**
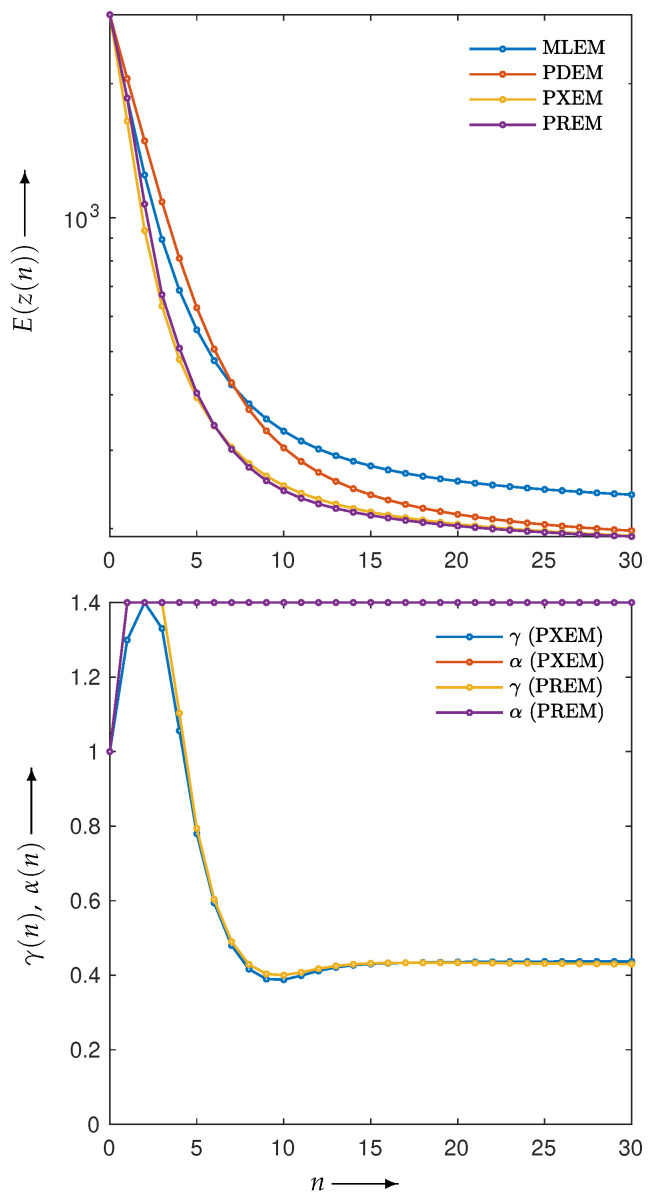
Change in evaluation function E(z(n)) with respect to the number of iterations *n* needed to reconstruct Shepp–Logan phantom images using MLEM, PDEM, PXEM, and PREM (**upper**), and plot of parameters γ(n) and α(n) for image reconstructions using PXEM and PREM (**lower**).

First, let us examine the results of the numerical experiments. The reconstruction was performed using the MLEM, PDEM, PXEM, and PREM methods on the Shepp–Logan phantom image in Figure 1a. Figure 3 (for n=0,1,2,…,N) plots the evaluation function E(z(n)) and the parameters (γ(n),α(n)) used in the PXEM and PREM methods versus the iteration number *n*. As indicated in the figure, PDEM had a lower evaluation function value than that of MLEM from the middle of the iterations onward, and the evaluation function value E(z(N)) at iteration *N* was significantly smaller than that of MLEM. This is because the fixed parameters of PDEM are chosen in a way that reduces the evaluation function at the maximum number of iterations *N*. On the other hand, PXEM and PREM showed smaller evaluation function values than those of PDEM or MLEM at all iteration numbers. As for the parameters, PXEM and PREM showed almost identical changes, indicating that the method using the reduced-size system worked as intended. Regarding the computational time needed for reconstruction, PDEM and MLEM each took approximately 14 s, while the additional time required to obtain the parameter sequence for each iteration using the reduced-size system was 4 s (approximately 30% more time than the computation at the original size). The parameter plot shows that α quickly reached an upper limit value, while γ starts out large at low iteration numbers and converges to around 0.4. This property is in accord with the results of previous studies [22] investigating the iteration numbers at which PDEM demonstrates high performance. Overall, these results confirm the effectiveness of the proposed methods, PXEM and PREM.

**Figure 4:**
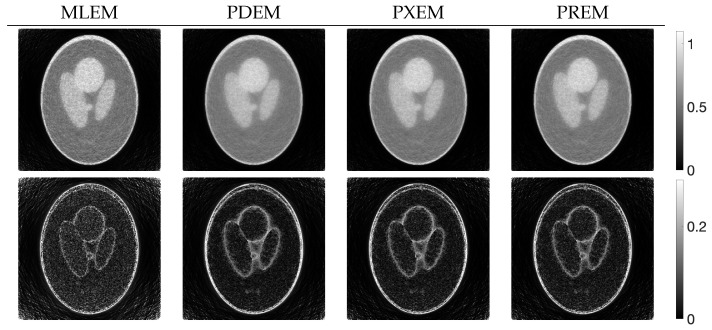
Images reconstructed from Shepp–Logan phantom images using MLEM, PDEM, PXEM, and PREM (**upper**) and their corresponding subtraction images (**lower**).

**Figure 5:**
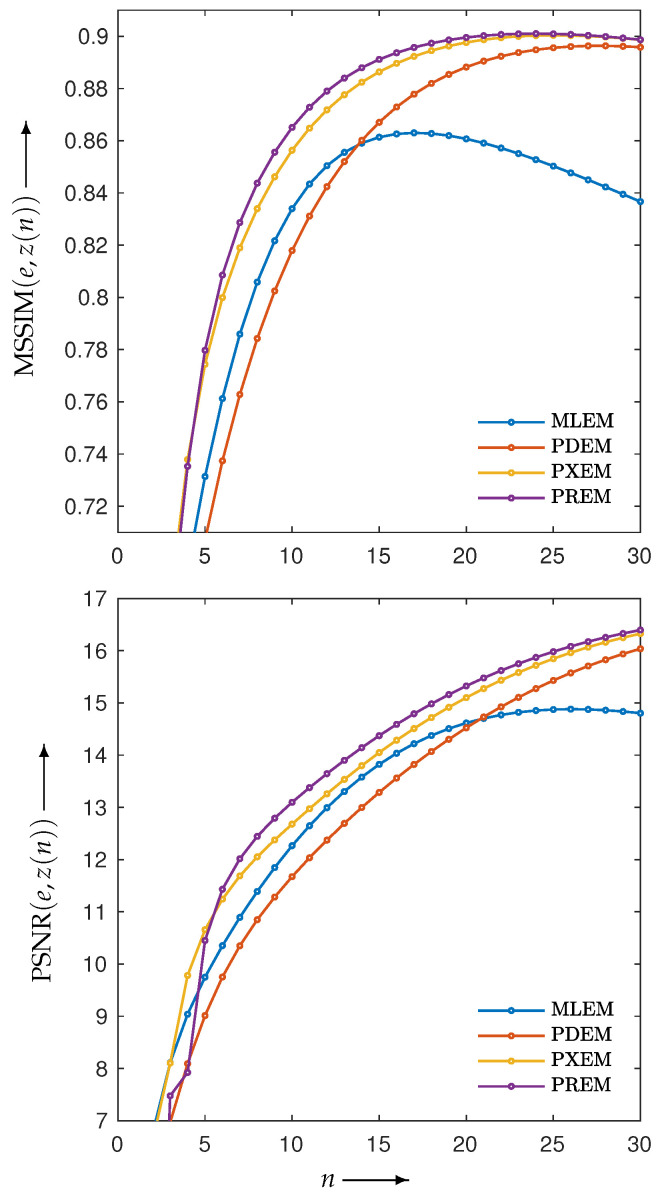
Change in MS-SSIM (MSSIM(e,z(n))) (**upper**) and PSNR (PSNR(e,z(n))) (**lower**) with respect to the number of iterations *n* needed to reconstruct image of Shepp–Logan phantom by using MLEM, PDEM, PXEM, and PREM.

Figure 4 shows the reconstructed images z(N) obtained by each iterative method, along with the absolute subtraction images from the true image. Each of the display ranges of the reconstructed images and the subtraction images are unified for the sake of comparison. The reconstruction produced by MLEM was affected by projection noise, whereas those produced by PDEM, PXEM, and PREM showed fewer effects of noise. The quality of the images reconstructed by PXEM and PREM, in terms of preserving edges, will be discussed by referring to results from other phantoms. The high quality of the images reconstructed by PXEM and PREM is also evident from the graphs of MS-SSIM and PSNR shown in Figure 5. Specifically, it is found that PXEM provides a better quantitative evaluation compared with MLEM and PDEM in all iterations and that PREM performs similarly to PXEM.

**Figure 6:**
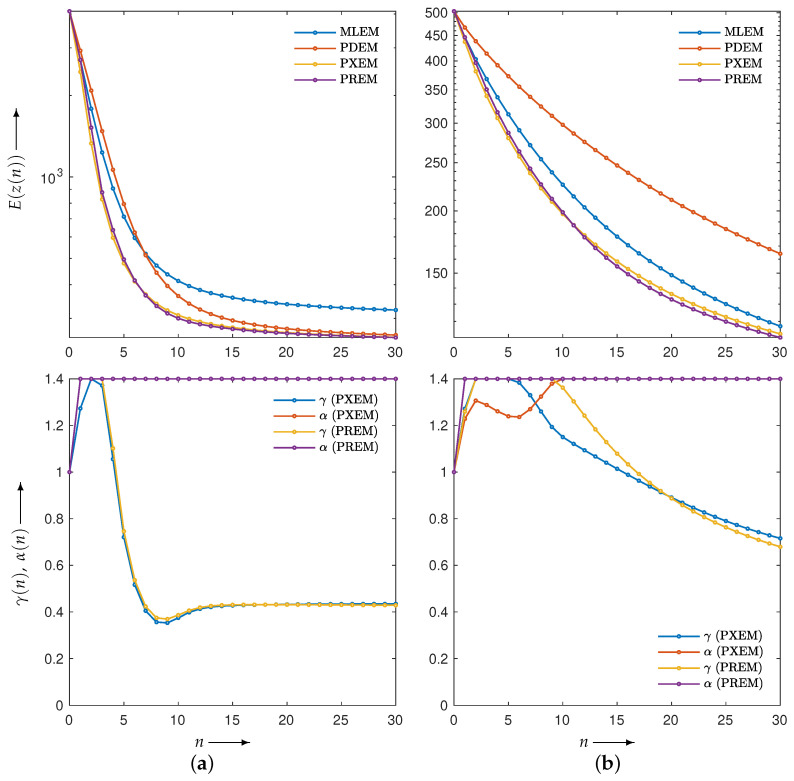
Change in evaluation function E(z(n)) with respect to the number of iterations *n* needed to reconstruct images of (**a**) disc and (**b**) chessboard phantoms by using MLEM, PDEM, PXEM, and PREM (**upper**), and plot of parameters γ(n) and α(n) for images reconstructed using PXEM and PREM (**lower**).

**Figure 7:**
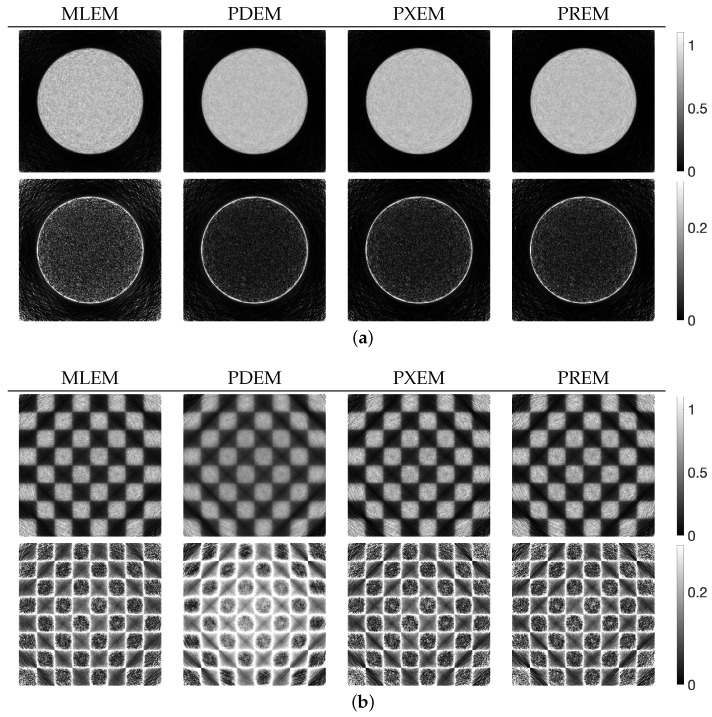
Images reconstructed from (**a**) disc and (**b**) chessboard phantom images using MLEM, PDEM, PXEM, and PREM (**upper**) and their corresponding subtraction images (**lower**).

**Figure 8:**
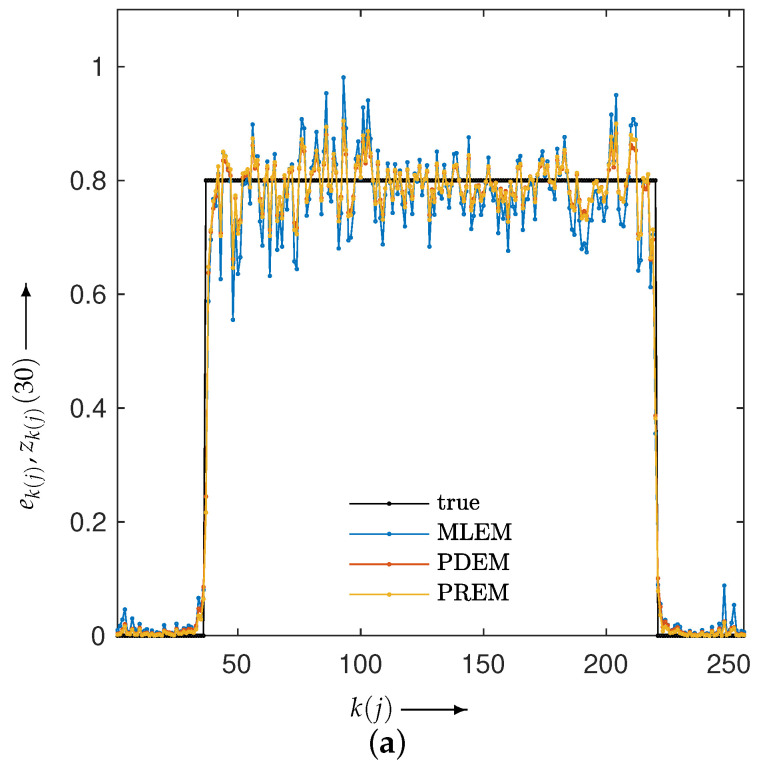

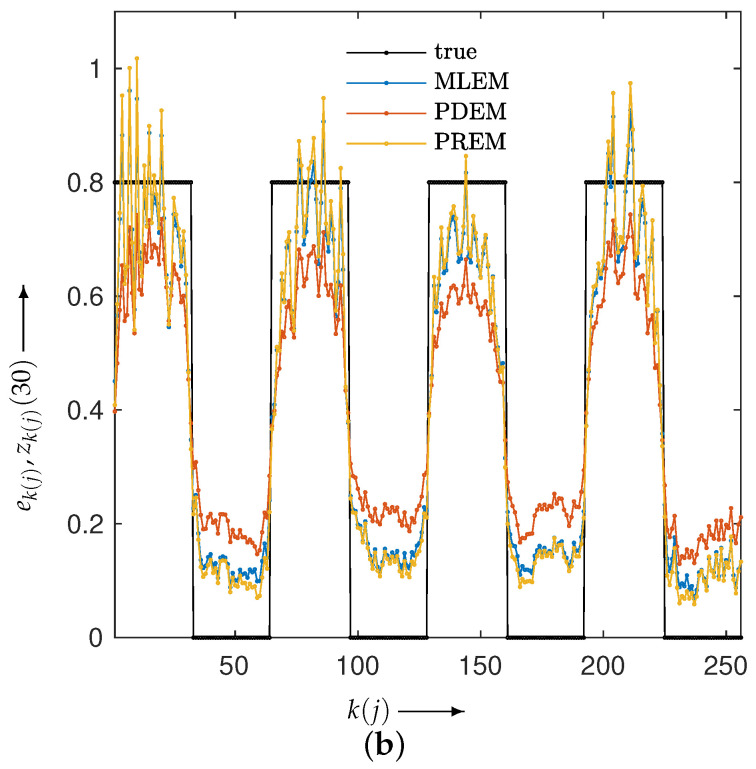
Density profiles along the column direction fixed at the 102nd row in the images reconstructed using MLEM, PDEM, and PREM for (**a**) disc and (**b**) chessboard phantoms.

We examined the properties of reconstruction for low-frequency and high-frequency images using disk and chessboard phantom images shown in Figure 1b and Figure 1c, respectively. Figure 6 plots the changes in the evaluation function and parameters. The results for the disc phantom were qualitatively similar to those obtained for the Shepp–Logan phantom, while the results for the chessboard phantom had different characteristics. This indicates that the proposed method should work appropriately not only for generic phantoms like the Shepp–Logan and disc phantoms, but also for geometric patterned images such as the chessboard. The reconstructed images and subtraction images at the maximum iteration number *N* are presented in Figure 7, and the density profiles in the column direction at the 102nd row of the images are shown in Figure 8. k(j) in the figure represents the index $k=1,2,\ldots ,J_{s}$ that satisfies the equation for the column of the 102nd row of pixels, which is given by $j=102+J_{s}\times \left(k-1\right)$. The low-frequency images reconstructed by PXEM and PREM show a similar reduction in the randomness of the density values in the flat regions as PDEM, whereas the reproducibility of the edges in the high-frequency images is comparable to that of MLEM, with a higher contrast compared with PDEM. This is evident not only visually but also quantitatively. Table 1 shows the standard deviation of the subtraction images in the reconstruction of the disc phantom and the contrast of the reconstructed images of the chessboard phantom. Contrast was determined as the difference between the mean high and low intensities. The results indicate that PXEM and PREM performed the parameter estimation appropriately so as to achieve an ideal standard deviation and contrast for objects that should exhibit both flat and steep characteristics.

**Figure 9:**
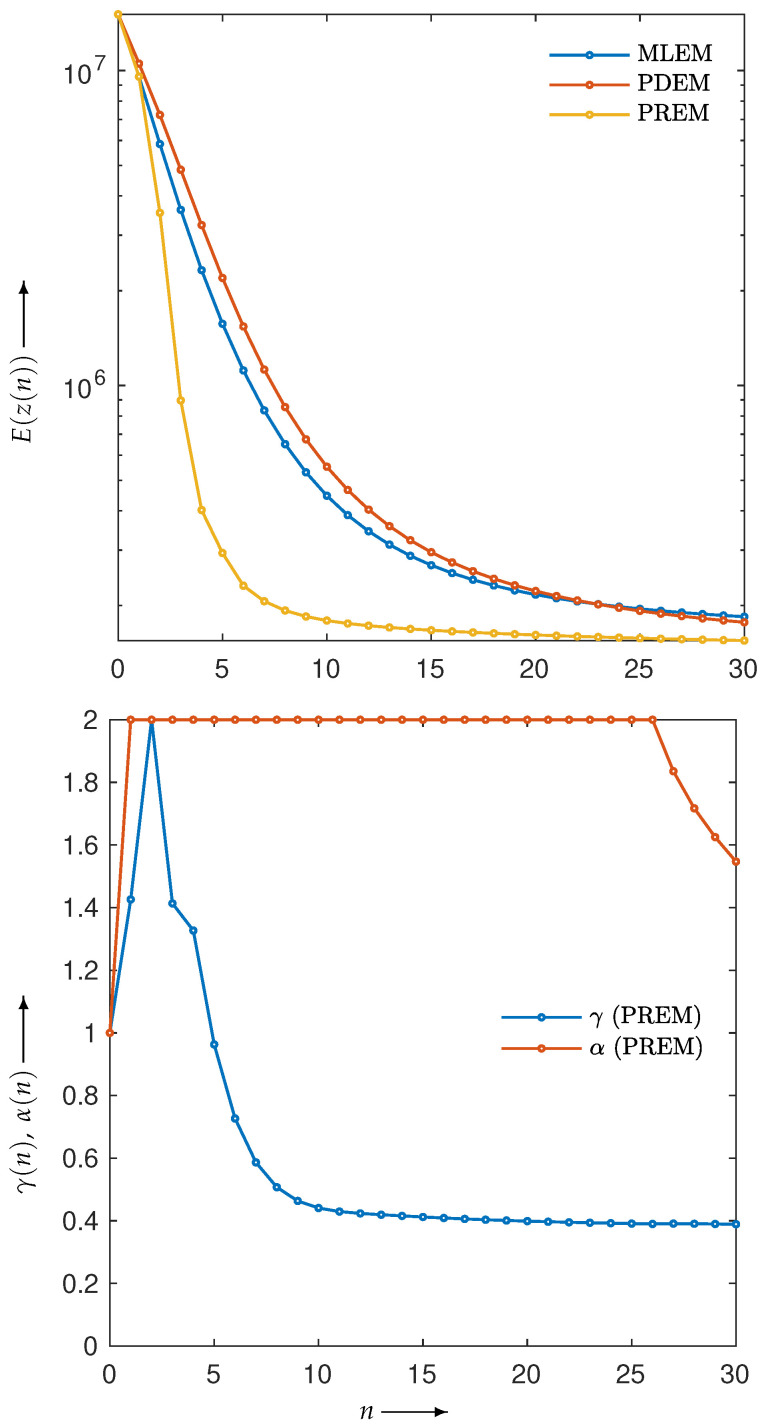
Change in evaluation function E(z(n)) with respect to the number of iterations, *n*, for sinogram obtained from X-ray CT scanner when using MLEM, PDEM, and PREM (**upper**), and plot of parameters γ(n) and α(n) for image reconstructed using PREM (**lower**).

**Figure 10:**
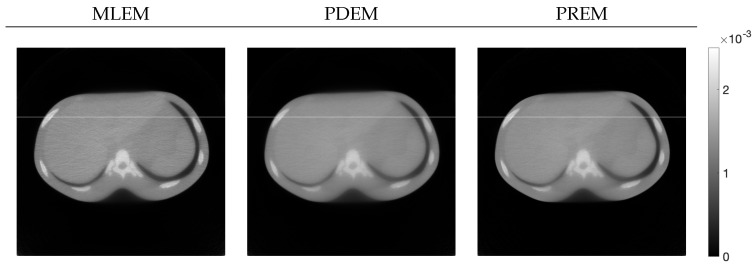
Images reconstructed from sinogram obtained from X-ray CT scanner by using MLEM, PDEM, and PREM (the white horizontal lines on the images indicate the positions of the density profiles in Figure 11).

**Figure 11:**
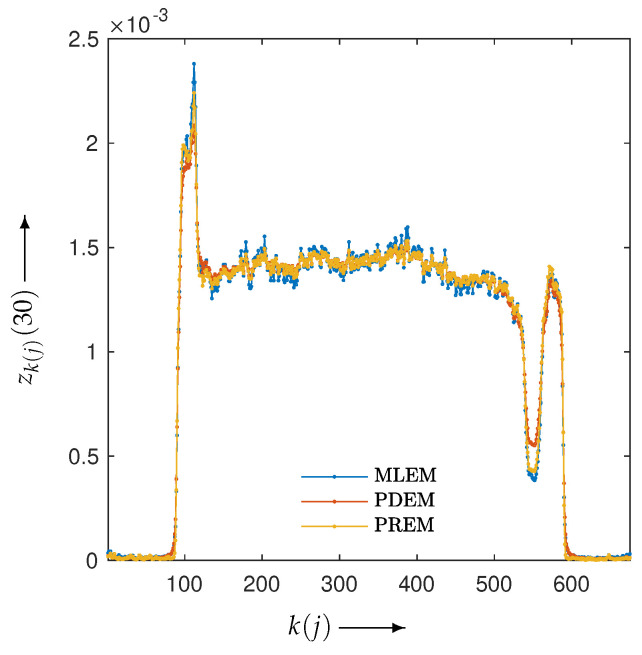
Density profiles along the lines indicated in Figure 10 in images reconstructed from sinogram obtained from X-ray CT scanner by using MLEM, PDEM, and PREM.

Next, let us examine the results of the physical experiment. The MLEM, PDEM, and PREM methods used the projections shown in the sinogram of Figure 2. The preprocessing time for the parameter estimation in PREM using the reduced-size system was 67 s, which represented an additional 32% of the reconstruction time compared with the original size, which took 207 s. Additionally, Figure 9 plots the changes in the evaluation function and parameters of PREM. Similarly to the numerical experiments, the proposed method operated appropriately. In the transition of the evaluation function, PREM significantly reduced the function value with fewer iterations compared with MLEM and PDEM. The reconstructed images and the density profile are shown in Figure 10 and Figure 11, respectively. The term k(j) in the figure denotes the index *k* on the white horizontal line in the reconstructed image $z_{j}$, where $k=1,2,\ldots ,J_{s}$. The profile shown in Figure 11, where there are gradual changes in density (in the range of indices k(j) from approximately 150 to 500) and sharp transitions (around index k(j) 550), indicates that PREM effectively suppressed artifacts caused by noise to a level comparable to PDEM, maintained sharp edges with the same contrast as MLEM, and combined the strengths of PDEM and MLEM.

Finally, we discuss the reason behind selecting the function in Equation (10) for parameter optimization. When the true image value *e* exists in the model of Equation (6) without noise and our aim is to reconstruct a high-quality image from the noisy projection Ae+σ based on the optimization principle, it is effective to choose an objective function as a function that directly utilizes the difference between the true and reconstructed images. However, in practice, it is necessary to represent the difference between the measured and forward projections using an objective function. When the inverse problem is ill-posed, selecting an effective objective function without using the true value becomes crucial for obtaining a more effective optimal solution.

Regarding the parameter optimization function Φ(z,γ,α) for the minimization problem in Equation (9), we considered not only the weighted EPD defined in Equation (10) concerning measured and forward projections, but also the KL-divergence

(14) $$\Phi \left(z,\gamma ,\alpha \right):=\sum_{i=1}^{I}\phi_{1,1}\left(y_{i},A_{i}f\left(z,\gamma ,\alpha \right)\right)$$

and the Euclidean distance

(15) $$\Phi \left(z,\gamma ,\alpha \right):={∥y-Af(z,\gamma ,\alpha )∥}_{2}.$$

We compared these with the Euclidean distance between the true and reconstructed images,

(16) $$\Phi \left(z,\gamma ,\alpha \right):={∥e-f(z,\gamma ,\alpha )∥}_{2}.$$

**Figure 12:**
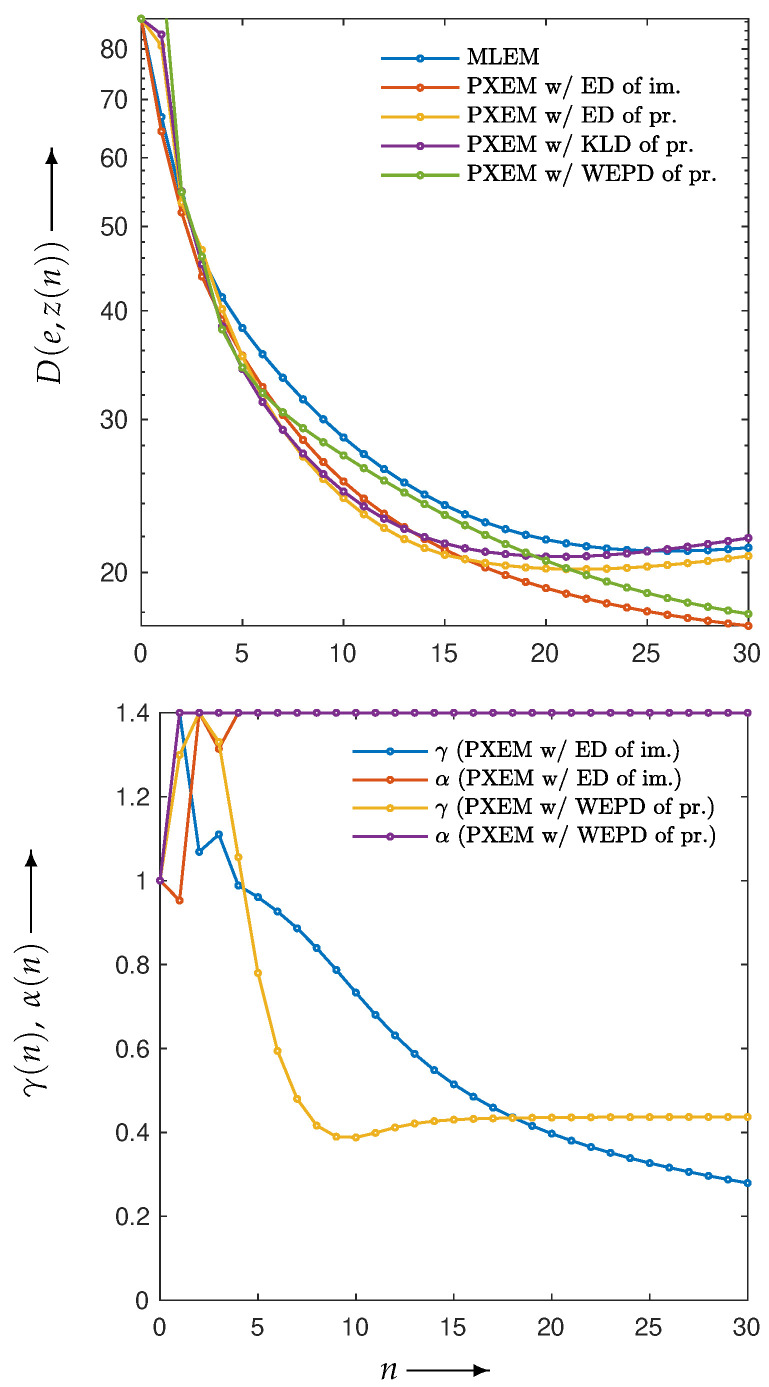
Change in D(e,z(n)) with respect to the number of iterations *n* needed to reconstruct Shepp–Logan phantom using MLEM and PXEM with parameter optimization functions of Euclidean distance (ED) of images, ED of projections, KL-divergence (KLD) of projections, and weighted extended power divergence (WEPD) of projections (**upper**), and plot of parameters γ(n) and α(n) for reconstruction using PXEM with Euclidean distance (ED) of images and weighted extended power divergence (WEPD) of projections (**lower**).

**Figure 13:**
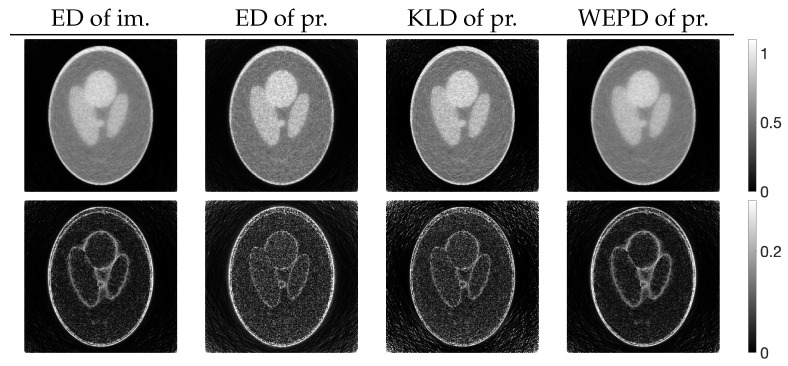
Images reconstructed from Shepp–Logan phantom using PXEM with parameter optimization functions of Euclidean distance (ED) of images, ED of projections, KL-divergence (KLD) of projections, and weighted extended power divergence (WEPD) of projections (**upper**) and their subtraction images (**lower**).

**Figure 14:**
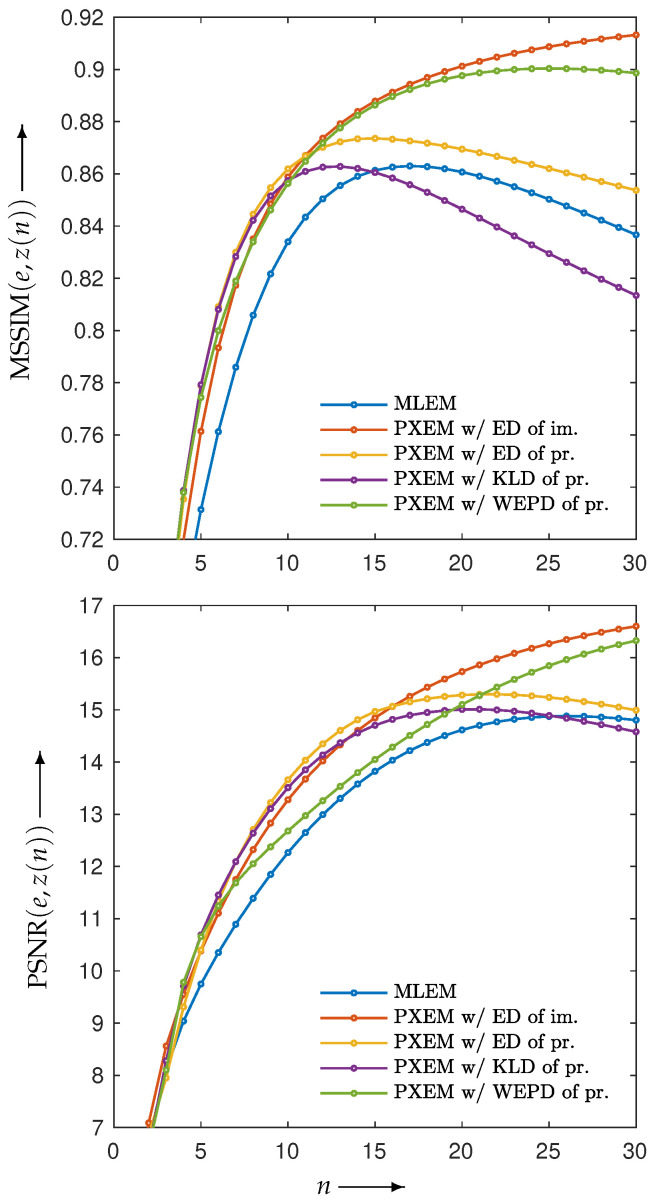
Change in MS-SSIM (MSSIM(e,z(n))) (**upper**) and PSNR (PSNR(e,z(n))) (**lower**) with respect to the number of iterations *n* needed to reconstruct Shepp–Logan phantom image by using MLEM and PXEM with parameter optimization functions of Euclidean distance (ED) of images, ED of projections, KL-divergence (KLD) of projections, and weighted extended power divergence (WEPD) of projections.

Using these four functions, reconstructions were performed with PXEM. Figure 12 (upper) shows the transition of the Euclidean distance

(17) $$D\left(e,z\left(n\right)\right):={∥e-z(n)∥}_{2},$$

for n=0,1,2,…,N, of images reconstructed by using PXEM with the parameter optimization functions defined in Equation (10) and Equations (14)–(16) as well as images using MLEM for comparison. Figure 12 (lower) plots the parameter changes for Equations (10) and (16). In the upper part of Figure 12, it is a well-known phenomenon that the function *D* with MLEM increases with a large number of iterations due to the relatively heavy noise added to the projections. On the other hand, with PXEM using Equation (16) based on the true image, the function value monotonically decreases as the number of iterations increases. Comparing the results of Equations (10), (14), and (15), which are functions of the measured and forward projections, reveals that only the case of Equation (10) exhibits a monotonic decrease. A visual assessment of the quality of the reconstructed images in Figure 13, as well as the evaluations using MS-SSIM and PSNR in Figure 14, confirms the superiority of the approach using Equation (10) for parameter optimization.

Moreover, the reason for setting the parameters $\left(\gamma^{0},\alpha^{0}\right)$ in Equation (10) to (0.5,1.2) is twofold. Firstly, fixed values help to demonstrate that PXEM and PREM algorithms operate effectively without fine-tuning. The second reason is based on Proposition 1, which guarantees the upper limit provided by the Hellinger distance with excellent robustness properties [33,34], and previous studies showing that similar values of the parameters $\left(\gamma^{0},\alpha^{0}\right)$ in the PDEM algorithm yielded high performance [22,35].

## 4. Conclusions

We proposed an iterative image reconstruction algorithm utilizing dynamic parameter tuning based on optimization principles, referred to as PXEM. We also devised a practical version of PXEM, called the PREM approach, where the preprocessing of parameter estimation is performed by reducing the image size and projection directions, thereby reducing computational costs. In numerical and physical experiments, these algorithms were found to combine the advantages of PDEM in suppressing the effects of measurement noise and the performance of MLEM in edge preservation. The underlying principle of the proposed method lies in the discovery of an extended power divergence, weighted by the projection operator. This extended power divergence, serving as a function of the measured and forward projections, demonstrates performance comparable to parameter optimization functions that use the difference between true and reconstructed images. The evaluation and analysis of the extended power divergence, including the rationale behind the choice of the fixed parameters $\left(\gamma^{0},\alpha^{0}\right)=\left(0.5,1.2\right)$, are not limited to tomographic image reconstruction problems; they have broader implications for optimization problems in general. In the numerical experiments, almost-identical parameter sequences were obtained for different phantoms. This suggests that additional time for parameter estimation using a new procedure may not be required if the conditions for projections that yield similar parameter sequences are the same. Further investigations into the conditions for the parameter sequences is a task for future research.

In regard to future applications, this model holds promise in enhancing medical imaging diagnostics, where precise image reconstruction under noisy conditions is critical. Additionally, its adaptive parameter tuning capability suggests potential applications in real-time imaging systems for optimizing performance across diverse imaging modalities.

## Figures and Tables

**Table 1:** Standard deviation and contrast obtained from the reconstruction of the disc and chessboard phantom images using the MLEM, PDEM, PXEM, and PREM.

	MLEM	PDEM	PXEM	PREM
std. dev.	0.083	0.056	0.056	0.057
contrast	0.532	0.418	0.544	0.550

## Data Availability

All data used to support the findings of this study are included within the article.
